# Inequalities in the burden of female breast cancer in Brazil, 1990–2017

**DOI:** 10.1186/s12963-020-00212-5

**Published:** 2020-09-30

**Authors:** Maximiliano Ribeiro Guerra, Mário Círio Nogueira, Deborah Carvalho Malta, Camila Soares Lima Côrrea, Maria de Fatima Marinho de Souza, Maria Paula Curado, Mariana Santos Felisbino-Mendes, Meghan Mooney, Mohsen Naghavi, Maria Teresa Bustamante-Teixeira

**Affiliations:** 1 Graduate Program in Public Health, Universidade Federal de Juiz de Fora (UFJF), Juiz de Fora, Minas Gerais Brazil; 2 Department of Maternal and Child Nursing and Public Health, Nursing School, Universidade Federal de Minas Gerais (UFMG), Belo Horizonte, Minas Gerais Brazil; 3 Graduate Program of the Preventive Medicine Department, Medical School, Universidade Federal de Minas Gerais (UFMG), Belo Horizonte, Minas Gerais Brazil; 4 AC Camargo Cancer Center, AC Camargo Hospital, São Paulo, SP Brazil; 5 Institute for Health Metrics and Evaluation (IHME), University of Washington, Seattle, WA USA

**Keywords:** Breast neoplasm, Incidence, Mortality, Global burden

## Abstract

**Background:**

Breast cancer is the most frequently diagnosed cancer in women and the leading cause of cancer death among females worldwide. In recent decades, breast cancer death rates have been stable or decreasing in more developed regions; however, this has not been observed in less developed regions. This study aims to evaluate inequalities in the burden of female breast cancer in Brazil including an analysis of interregional and interstate patterns in incidence, mortality and disability-adjusted life years (DALYs) rates from 1990 to 2017, and mortality-to-incidence ratio (MIR), and their association with the Socio-demographic Index (SDI).

**Methods:**

Using estimates from the global burden of disease (GBD) study, we applied a spatial exploratory analysis technique to obtain measurements of global and local spatial correlation. Percentage changes of breast cancer incidence, mortality, and DALYs rates between 1990 and 2017 were calculated, and maps were developed to show the spatial distribution of the variables. Spatial panel models were adjusted to investigate the association between rates and SDI in Brazilian states.

**Results:**

In Brazil, while breast cancer mortality rate have had modest reduction (−4.45%; 95% UI: −6.97; −1.76) between 1990 and 2017, the incidence rate increased substantially (+39.99%; 95% UI: 34.90; 45.39). Breast cancer incidence and mortality rates in 1990 and 2017 were higher in regions with higher SDI, i.e., the most developed ones. While SDI increased in all Brazilian states between 1990 and 2017, notably in less developed regions, MIR decreased, more notably in more developed regions. The SDI had a positive association with incidence rate and a negative association with MIR.

**Conclusion:**

Such findings suggest an improvement in breast cancer survival in the period, which may be related to a broader access to diagnostic methods and treatment. This study also revealed the inequality in breast cancer outcomes among Brazilian states and may guide public policy priorities for disease control in the country.

## Background

Breast cancer is the most common type of cancer and the main cause of cancer-related death among females worldwide [1–3], as well as the leading cause of disability-adjusted life years (DALYs) due to cancer in women [3]. Although breast cancer incidence rates are higher in more developed regions, the incidence in less developed regions has increased in the last decades, while it has remained relatively stable or even reduced in more developed regions [3, 4]. Likewise, breast cancer death rates have been decreasing in many high-income countries, while mortality rates continue to increase in several low- and middle-income countries, such as those in Latin America and the Caribbean, and parts of Asia [4].

In general, the range in mortality rates between world regions is lower than for incidence rates as a result of better survival of breast cancer in developed regions. The use of the mortality-to-incidence ratio (MIR), in this context, can allow a better identification of the heterogeneity in breast cancer burden [5].

Nevertheless, even within regions, there may be remarkable geographic variability in disease incidence and mortality patterns. In Central and South America, for instance, there was a wide variation in breast cancer rates across the region, with the highest rates in Central America almost 50% lower than the highest rates observed in South America. Breast cancer incidence in Argentina, Brazil, and Uruguay, for instance, reached levels similar to those of other countries of very high economic development, such as the USA and Canada. On the other hand, while Uruguay and Argentina had some of the highest breast cancer mortality rates in the world, Guatemala and Nicaragua had the lowest rates [6].

In Brazil, breast cancer incidence increased from an age-standardized rate of 54.73 (95% U.I.: 45.52‑60.30) in 1990 to 74.02 (95% U.I.: 61.90‑85.86) in 2015 [7]. While breast cancer incidence has increased in Brazil, there have been conflicting reports as to if breast cancer mortality has remained stable [7–9] or increased [10–14]. However, all studies have shown regional disparities in breast cancer mortality in the Brazilian regions, usually with rates remaining stable or declining in more developed regions and increasing in less developed regions [6, 8, 10, 11, 15, 16].

Regional variation in breast cancer rates over time most likely reflects changes and differences in multiple factors including the availability of early detection and healthcare access, the prevalence of physical inactivity, alcohol consumption and excess weight, and variability in registration practices [2, 4, 6, 17]. Given these known factors that contribute to the global burden of breast cancer, studies that are assessing breast cancer in a given region over time should always consider the possibility of intra and interregional variation, especially in regions with limited health resources [18]. In addition, the use of maps to identify the areas of greater risk, taking into account the disease control network, can be used to outline strategies to reduce the magnitude of breast cancer on the local population.

This study aims to evaluate inequalities in the burden of breast cancer in Brazil by means of the analysis of interregional and interstate variation in incidence, mortality and DALYs rates from 1990 to 2017, and MIR, and their association with the socio-demographic index (SDI).

## Methods

### Data sources

All analyses were carried out using data of global burden of disease study 2017 (GBD 2017), coordinated by the Institute for Health Metrics and Evaluation (IHME) that estimated the burden of diseases, injuries, and risk factors for 195 countries and territories and at the subnational level for a subset of countries [19].

Input data to estimate breast cancer rates came from the Brazilian cancer registry for incidence data, and from the mortality information system of the Brazilian Ministry of Health for death data, with adjustment for underreporting of deaths, and for ill-defined/nonspecific causes, called garbage codes [20]. We used the IBGE (Brazilian Institute of Geography and Statistics) codes to show the spatial configuration of the Brazilian regions and states (Figure S1).

### Variables of interest

The outcome variables were rates of age-standardized breast cancer death (ASDR), incidence (ASIR) and disability-adjusted life year (DALYs) per 100,000 women, and mortality-to-incidence ratio (MIR). We reported 95% uncertainty intervals (95% UI) for mortality, incidence and DALYs rates, and respective percentual changes between 1990 and 2017 years.

The percentual changes were calculated as the proportional difference between rates from the year 2017 and the year 1990 in relation to the year 1990. In the GBD study methods, the uncertainty intervals are estimated systematically using Monte Carlo approach with the repetition of 1000 draws for each estimate. Thus, the 95% UIs are the 2·5th and 97·5th percentiles of the draw-level values are the lower and upper bound, respectively [21].

We divided publicly available data of all age-standardized death rates by all incidence rates of breast cancer in females from the GBD 2017 study to calculate the mortality-to-incidence ratio (MIR) [22].

The exposure variable was the socio-demographic index (SDI). Developed by GBD researchers, the socio-demographic index (SDI) is a measure of socio-demographic development of a geographic region, based on average income per person, educational attainment, and total fertility rate. It contains an interpretable scale in which zero (0) represents the lowest socio-demographic development observed across all GBD geographies from 1970 to 2017, and one (1) represents the opposite, i.e., the highest SDI for the same period [23].

All estimates were made for Brazil and states (i.e., the Federative Units - FU) from 1990 to 2017. All variables were grouped into quintiles in order to visualize its spatial distribution in Brazilian states based on its value for years 1990 and 2017. All estimates and their respective percentage variation were shown in tables and maps. Temporal distribution plots of indicators over the considered period were made for Brazil and states.

### Statistical analyses

For exploratory analysis, scatter plots were used, and Pearson correlation coefficients between the variables were estimated. We estimated a measure of global spatial autocorrelation, the Moran I coefficient, which evaluates whether the measure of the variable in one region is correlated with the measure of the same variable in neighboring regions. We also estimated a local spatial correlation, the LISA estimator [24], and from its results were created maps of local clusters, that is, groups of regions with values for a variable significantly similar to their neighbors. The clusters are called high-high and low-low, respectively for the cluster of values above and below the average of all regions. In order to elaborate the spatial weights, the criterion of four nearest neighbors was used, after estimations with several options to verify which neighborhood matrix captured more the spatial dependence.

To investigate the relationship between SDI and the other variables in Brazilian states from 1990 to 2017, panel data models were adjusted. The Hausman test [25] was used to guide the choice between fixed or random-effects model, and the Pesaran test [26] to evaluate the presence of cross-sectional dependence in observations, which would indicate the need to adjust for the spatial correlation by spatial panel models. Spatial models known as SAR (spatial auto-regressive) include a spatial lag of the dependent variable as covariate.

All analyzes were performed in the R statistical program [27], using the suggestions of packages and functions for spatial analysis from Anselin [28].

## Results

**Fig. 1:**
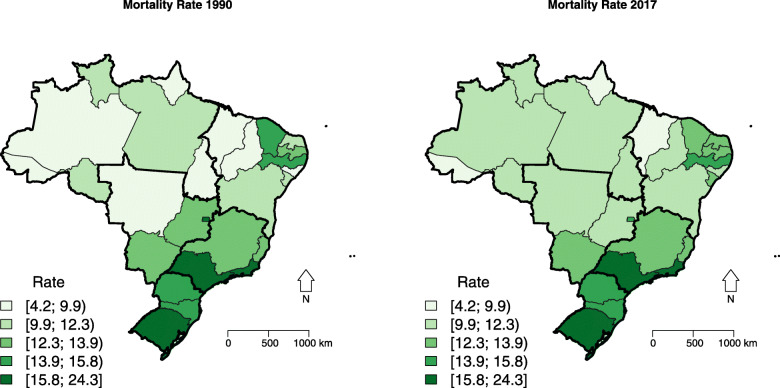
Spatial distribution of age-standardized female breast cancer death (ASDR) rate in Brazilian states, 1990 and 2017

For Brazil as a whole, breast cancer mortality rates ranged from 15.28 (95% UI: 14.95; 15.58) in 1990 to 14.60 (95% UI: 14.23; 14.97), with a decrease of 4.45% (95% UI: −6.97; −1.76) between 1990 and 2017 years. However, there was a geographical variation in mortality rates. Although in most states, death rates were stable in the period, some states had an increase in mortality, mainly in the Northeast region, with the largest increases in the states of Rio Grande do Norte (+27.56%; 95% UI: 13.04; 44.00) and Alagoas (+28.97%; 95% UI: 12.37; 47.11). Nearly half of the states in the South, Southeast, and Central-West regions had a reduction in mortality rates in the period, with the largest reductions in the states of Rio Grande do Sul (−14.08%; 95% UI: −20.22; −7.54), São Paulo (−14.26%; 95% UI: −19.84; −8.73), and Distrito Federal (−14.71%; 95% UI: −22.66; −5.40) (Table 1; Figs. 1 and S2).

**Table 1:** Age-standardized female breast cancer death (ASDR), incidence (ASIR) and disability-adjusted life years (DALYs) rates according to Brazilian states and regions in 1990 and 2017, and percentage change (Δ%)

		ASDR (UI 95%)			ASIR (UI 95%)			DALY (UI 95%)		
IBGE CODE	REGION/UF	1990	2017	Δ%	1990	2017	Δ%	1990	2017	Δ%
-	**Brazil**	15.28	14.60	−4.45	29.75	41.65	39.99	440.44	428.37	−2.74
		14.95-15.58	14.23; 14.97	−6.97; −1.76	29.00; 30.48	40.22; 43.13	34.90; 45.39	430.03; 450.96	415.39; 442.43	−5.72; 0.44
1	**North region**									
11	**Rondônia**	10.05	10.51	4.62	17.90	28.19	57.55	294.76	307.46	4.31
		9.01; 11.11	9.01; 12.17	−12.02; 24.77	15.83; 20.01	23.91; 32.90	29.80; 89.22	263.37; 329.48	260.63; 357.79	−13.79; 24.66
12	**Acre**	8.59	9.14	6.34	15.30	21.90	43.11	256.05	261.43	2.10
		7.98; 9.25	8.36; 9.95	−5.58; 18.96	14.10; 16.68	19.68; 24.16	24.76; 63.17	236.28; 277.19	237.09; 285.87	−10.34; 15.60
13	**Amazonas**	9.69	11.60	19.64	17.80	30.39	70.77	287.14	345.43	20.30
		8.87; 10.52	10.67; 12.50	7.02; 34.76	16.18; 19.45	27.45; 33.49	49.70; 95.09	261.73; 313.59	317.05; 375.15	6.76; 36.09
14	**Roraima**	12.14	11.57	−4.68	21.32	28.27	32.64	328.78	305.37	−7.12
		10.69; 13.73	9.69; 13.50	−23.57; 17.98	18.55; 24.43	23.48; 33.40	5.12; 68.19	283.97; 373.73	253.42; 360.18	−26.34; 16.70
15	**Pará**	10.03	10.31	2.73	18.55	25.53	37.64	303.22	304.74	0.50
		9.28; 10.83	9.52; 11.10	−7.63; 14.29	16.82; 20.32	23.16; 28.03	21.70; 56.00	278.48; 328.75	279.40; 328.33	−10.33; 12.58
16	**Amapá**	8.63	9.59	11.13	16.76	24.57	46.58	252.75	277.91	9.95
		7.94; 9.34	8.77; 10.42	−0.78; 24.82	15.24; 18.39	22.30; 27.15	28.55; 67.05	231.80; 275.31	253.65; 304.13	−2.76; 24.22
17	**Tocantins**	9.60	10.06	4.83	18.43	26.78	45.26	269.92	299.59	10.99
		8.21; 11.01	9.10; 11.03	−11.76;25.29	15.23; 21.40	23.67; 29.98	20.24; 77.49	222.17; 316.32	269.37; 330.84	−8.12; 35.35
2	**Northeast region**									
21	**Maranhão**	7.67	9.22	20.33	13.30	21.69	63.02	221.06	273.68	23.81
		6.51; 9.02	8.41; 10.03	0.90; 43.88	11.04; 15.77	19.49; 23.78	33.53; 98.71	185.99; 260.07	249.38; 298.48	2.62; 50.43
22	**Piauí**	9.08	10.26	12.97	18.23	25.93	42.27	277.06	313.61	13.19
		8.11; 10.20	9.43; 11.13	−2.51; 30.83	16.07; 20.69	23.49; 28.49	20.94; 67.69	245.43; 313.69	286.57; 340.58	−2.83; 32.68
23	**Ceará**	14.57	13.30	−8.74	28.83	34.25	18.81	443.67	396.94	−10.53
		13.04; 16.14	12.44; 14.20	−19.94;3.44	25.53; 32.29	31.33; 37.36	2.32; 38.40	395.69; 495.24	369.18; 427.86	−21.76; 1.88
24	**Rio Grande do Norte**	10.39	13.26	27.56	19.99	36.38	82.01	304.96	395.85	29.80
		9.39; 11.41	12.30; 14.27	13.04; 44.0	17.80; 22.28	33.02; 39.74	58.55; 109.89	272.42; 337.04	364.67; 431.10	13.76; 47.56
25	**Paraíba**	12.83	12.71	−0.93	24.40	33.00	35.22	379.53	375.08	−1.17
		11.62; 14.17	11.35; 14.22	−14.72; 16.15	21.74; 27.07	29.04; 37.18	13.86; 60.02	340.87; 420.58	330.96; 423.68	−15.19; 16.98
26	**Pernambuco**	14.84	15.31	3.13	25.93	37.79	45.71	432.17	448.36	3.75
		14.02; 15.72	14.37; 16.30	−5.30; 12.09	24.16; 27.76	34.69; 41.01	30.40; 62.23	404.79; 460.62	418.13; 480.12	−5.04; 13.82
27	**Alagoas**	8.99	11.60	28.97	15.04	28.20	87.52	266.13	347.90	30.73
		8.26; 9.88	10.68; 12.58	12.37; 47.11	13.63; 16.68	25.74; 30.96	61.97; 116.65	242.16; 295.02	318.95; 379.90	14.14; 50.31
28	**Sergipe**	11.53	13.56	17.63	20.63	34.74	68.40	331.69	404.14	21.84
		10.53; 12.48	12.50; 14.65	4.73; 30.85	18.80; 22.52	31.42; 37.97	48.06; 90.93	301.80; 361.61	370.64; 438.10	7.35; 37.39
29	**Bahia**	10.39	12.12	16.69	19.00	31.75	67.06	311.06	378.90	21.81
		9.47; 11.30	11.43; 12.96	5.19; 30.25	17.22; 20.77	29.24; 34.63	47.30; 89.98	283.17; 338.92	353.84; 407.68	9.09; 37.24
3	**Southeast region**									
31	**Minas Gerais**	12.75	13.18	3.42	23.71	38.03	60.41	364.47	398.10	9.23
		12.14; 13.33	12.40; 13.94	−3.48; 10.85	22.25; 25.12	35.11; 41.10	45.29; 76.40	345.73; 383.72	372.63; 424.72	0.99; 17.87
32	**Espírito Santo**	12.53	12.34	−1.57	23.77	34.96	47.06	357.71	368.92	3.14
		11.84; 13.27	11.43; 13.22	−10.25;7.26	22.16; 25.50	31.70; 38.23	31.21; 63.84	336.58; 381.76	340.48; 399.47	−6.95; 12.92
33	**Rio de Janeiro**	21.50	19.84	−7.73	41.54	56.72	36.53	608.30	579.95	−4.66
		20.65; 22.40	18.65; 21.06	−14.01; −0.69	39.30; 44.13	52.37; 61.55	24.09; 50.45	581.98; 636.69	542.42; 617.83	−11.54; 2.94
35	**São Paulo**	18.73	16.06	−14.26	37.88	49.96	31.88	528.36	466.40	−11.73
		18.03; 19.41	15.24; 16.90	−19.84; −8.73	35.87; 40.10	46.20; 54.24	20.41; 45.69	507.34; 551.97	439.47; 496.48	−17.90; −5.42
4	**South region**									
41	**Paraná**	14.50	15.41	6.28	27.28	43.94	61.04	403.36	447.12	10.85
		13.85; 15.21	14.52; 16.36	−1.25; 14.29	25.65; 28.98	40.40; 47.81	46.76; 78.62	383.92; 424.23	417.06; 478.25	2.03; 19.81
42	**Santa Catarina**	14.59	15.24	4.45	28.79	47.27	64.16	404.16	448.67	11.01
		13.91; 15.33	14.31; 16.31	−4.40;13.38	26.99; 30.68	43.45; 51.57	48.05; 83.55	382.13; 425.23	417.09; 482.49	1.31; 21.39
43	**Rio Grande do Sul**	20.41	17.54	−14.08	41.62	52.77	26.80	573.63	503.47	−12.23
		19.62;21.24	16.50;18.62	−20.22; −7.54	39.23; 44.09	48.53; 57.58	14.58; 40.22	548.26; 599.47	469.51; 538.79	−18.94; −5.03
5	**Central-West region**									
50	**Mato Grosso do Sul**	12.70	13.60	7.09	23.93	36.79	53.77	366.29	398.71	8.85
		11.87; 13.57	12.67; 14.71	−2.63; 18.38	22.14; 25.91	33.63; 40.43	36.71; 73.27	340.52; 393.30	369.65; 431.17	−1.95; 21.26
51	**Mato Grosso**	9.50	11.03	16.07	18.11	29.79	64.48	284.19	323.36	13.79
		8.50; 10.55	10.10; 11.99	1.43; 34.21	16.10; 20.20	26.96; 33.00	40.79; 92.65	252.49; 316.43	295.46; 352.88	−1.00; 32.40
52	**Goiás**	13.13	11.98	−8.75	23.91	32.96	37.83	366.05	357.80	−2.25
		12.37; 13.88	11.14; 12.86	−16.39; −0.45	22.19; 25.62	30.25; 36.09	24.20; 54.08	344.56; 387.99	332.50; 386.87	−11.26; 6.52
53	**Distrito Federal**	17.34	14.79	−14.71	36.71	49.83	35.76	478.06	393.29	−17.73
		16.32; 18.40	13.62; 16.02	−22.66; −5.40	34.17; 39.26	45.16; 55.15	20.83; 53.88	446.91; 508.18	361.20; 426.18	−25.29; −8.90

Rates per 100,000 inhabitants

*UI 95%* 95% uncertainty interval

**Fig. 2:**
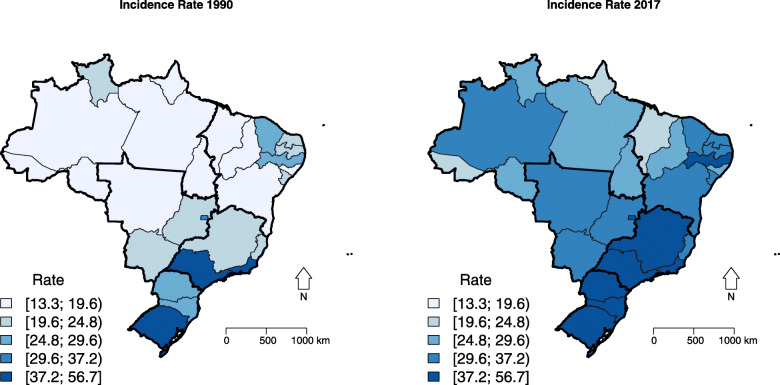
Spatial distribution of age-standardized female breast cancer incidence (ASIR) rate in Brazilian states, 1990 and 2017

Breast cancer incidence rates for Brazil in 1990 and 2017 were 29.75 (95% UI: 29.00; 30.48), and 41.65 (95% UI: 40.22; 43.13) new cases per 100,000 women, respectively, with an increase of 39.99% (95% UI: 34.90; 45.39) between 1990 and 2017 years. As well as for mortality, incidence rates were also higher in the Southeast and South regions, lower in the North and Northeast regions, and intermediate in the Central-West region. All Brazilian states showed an increase in incidence rate from 1990 to 2017, with a higher increase for some states of the North (Amazonas) and Northeast (Rio Grande do Norte, Alagoas, Sergipe, and Bahia) regions, which had lower values initially (Table 1; Figs. 2 and S2).

**Fig. 3:**
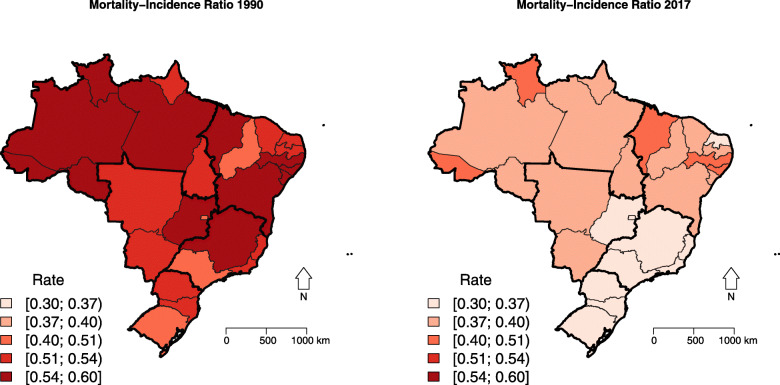
Spatial distribution of female breast cancer mortality-to-incidence (MIR) ratio in Brazilian states, 1990 and 2017

The MIR decreased in all states, more notably in the South and Southeast regions, in which it reached the lowest values in 2017 (Table 2; Figs. 3 and S3).

**Table 2:** Socio-demographic index (SDI) and female breast cancer mortality-to-incidence (MIR) ratio according to Brazilian states and regions in 1990 and 2017, and the percentage change (Δ%)

		SDI			MIR		
IBGE CODE	REGION/UF	1990	2017	Δ%	1990	2017	Δ%
-	Brazil	0.49	0.66	34.30	0.51	0.35	−31.75
1	North region						
11	Rondônia	0.42	0.62	46.82	0.56	0.37	−33.59
12	Acre	0.38	0.60	58.74	0.56	0.42	−25.69
13	Amazonas	0.44	0.63	42.81	0.54	0.38	−29.94
14	Roraima	0.43	0.65	50.09	0.57	0.41	−28.14
15	Pará	0.41	0.58	40.17	0.54	0.40	−25.36
16	Amapá	0.47	0.66	40.19	0.51	0.39	−24.19
17	Tocantins	0.40	0.61	53.29	0.52	0.38	−27.83
2	Northeast region						
21	Maranhão	0.31	0.51	60.71	0.58	0.43	−26.18
22	Piauí	0.37	0.55	49.93	0.50	0.40	−20.59
23	Ceará	0.41	0.60	44.89	0.51	0.39	−23.19
24	Rio Grande do Norte	0.42	0.61	44.92	0.52	0.36	−29.91
25	Paraíba	0.40	0.57	43.06	0.53	0.39	−26.73
26	Pernambuco	0.42	0.59	41.68	0.57	0.41	−29.22
27	Alagoas	0.36	0.56	55.26	0.60	0.41	−31.22
28	Sergipe	0.42	0.62	44.01	0.56	0.39	−30.15
29	Bahia	0.40	0.59	46.20	0.55	0.38	−30.15
3	Southeast region						
31	Minas Gerais	0.49	0.66	33.90	0.54	0.35	−35.53
32	Espírito Santo	0.50	0.68	34.91	0.53	0.35	−33.07
33	Rio de Janeiro	0.58	0.71	22.53	0.52	0.35	−32.42
35	São Paulo	0.56	0.72	28.54	0.49	0.32	−34.99
4	South region						
41	Paraná	0.51	0.68	32.39	0.53	0.35	−34.00
42	Santa Catarina	0.54	0.70	29.14	0.51	0.32	−36.37
43	Rio Grande do Sul	0.54	0.69	26.94	0.49	0.33	−32.24
5	Central-West region						
50	Mato Grosso do Sul	0.46	0.65	39.04	0.53	0.37	−30.36
51	Mato Grosso	0.47	0.66	38.73	0.52	0.37	−29.43
52	Goiás	0.46	0.65	40.62	0.55	0.36	−33.79
53	Distrito Federal	0.63	0.79	25.26	0.47	0.30	−37.18

**Fig. 4:**
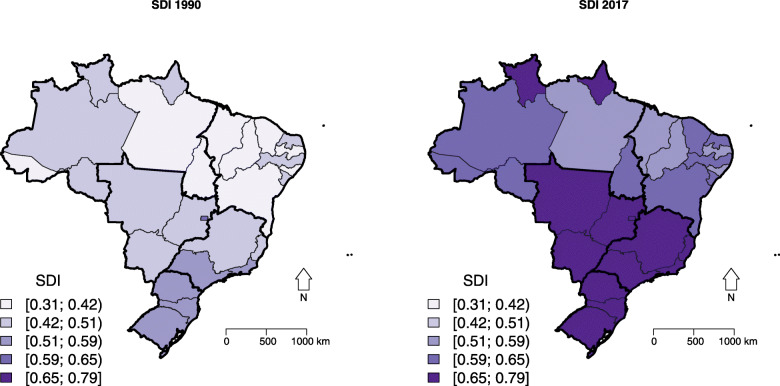
Spatial distribution of socio-demographic index (SDI) in Brazilian states, 1990 and 2017

Taking into account the Brazilian regions, SDI had higher values in the Southeast, South, and Central West, and lower values in the North and Northeast. From 1990 to 2017, SDI exhibited a steady increase in all states, although this increase was higher for states of the North and Northeast regions, which had the lowest values in 1990 (Table 2; Figs. 4 and S3).

**Fig. 5:**
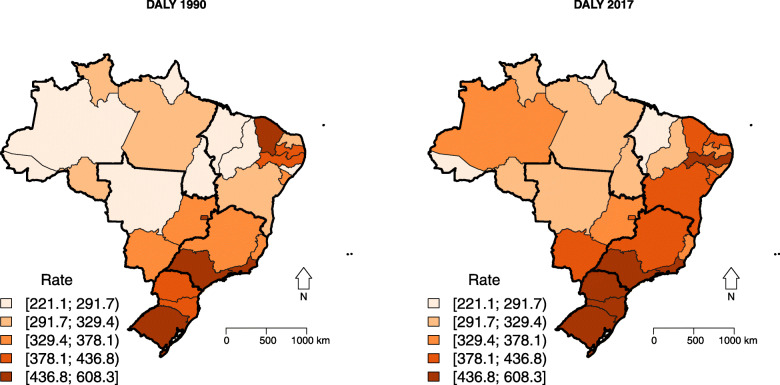
Spatial distribution of female breast cancer disability-adjusted life years (DALYs) rate in Brazilian states, 1990 and 2017

The DALYs rates had similar behavior to mortality, not only with higher values in the Southeast, South, and Central-West regions, but also with the greatest reductions in these regions between 1990 and 2017 years. However, only three states had a reduction in the period: Distrito Federal (−17.73%; 95% UI: −25.29; −8.90), Rio Grande do Sul (−12.23%; 95% UI: −18.94; −5.03), and São Paulo (−11.73%; 95% UI: −17.90; −5.42) (Table 1; Figs. 5 and S4).

In the exploratory analyzes, there was a significant correlation between all the variables both in 1990 and 2017 years. The SDI had a positive correlation with ASDR, ASIR, and DALYs, and negative correlation with MIR in 1990 and 2017 years (Table 3). Almost all the indicators exhibited significant spatial autocorrelation, except for MIR in 1990 (Table 4).

**Table 3:** Pearson correlation coefficient between socio-demographic index (SDI), age-standardized female breast cancer death (ASDR), incidence (ASIR) and disability-adjusted life years (DALYs) rates and female breast cancer mortality-to-incidence (MIR) ratio, Brazil, 1990 and 2017

Year	Variables	SDI	ASDR	ASIR	DALY	MIR
1990	SDI	1	0.79	0.82	0.76	−0.68
	ASDR	0.79	1	0.99	1.00	−0.52
	ASIR	0.82	0.99	1	0.99	−0.63
	DALY	0.76	1.00	0.99	1	−0.52
	MIR	−0.68	−0.52	−0.63	−0.52	1
2017	SDI	1	0.61	0.76	0.53	−0.87
	ASDR	0.61	1	0.96	0.99	−0.65
	ASIR	0.76	0.96	1	0.93	−0.82
	DALY	0.53	0.99	0.93	1	−0.61
	MIR	−0.87	−0.65	−0.82	−0.61	1

All *p* values < 0.05

**Table 4:** Spatial autocorrelation coefficients (Moran’s I) for socio-demographic index (SDI), age-standardized female breast cancer death (ASDR), incidence (ASIR) and disability-adjusted life years (DALYs) rates and female breast cancer mortality-to-incidence (MIR) ratio, Brazilian states, 1990 and 2017

Variables	Year	Moran’s I	*p* value*
SDI	1990	0.581	0.001
	2017	0.579	0.001
ASDR	1990	0.424	0.001
	2017	0.496	0.001
ASIR	1990	0.436	0.001
	2017	0.581	0.001
DALY	1990	0.387	0.001
	2017	0.525	0.001
MIR	1990	0.156	0.052
	2017	0.577	0.001

*p value obtained per 1000 Monte-Carlo simulations; significant if *p* < 0.05

**Fig. 6:**
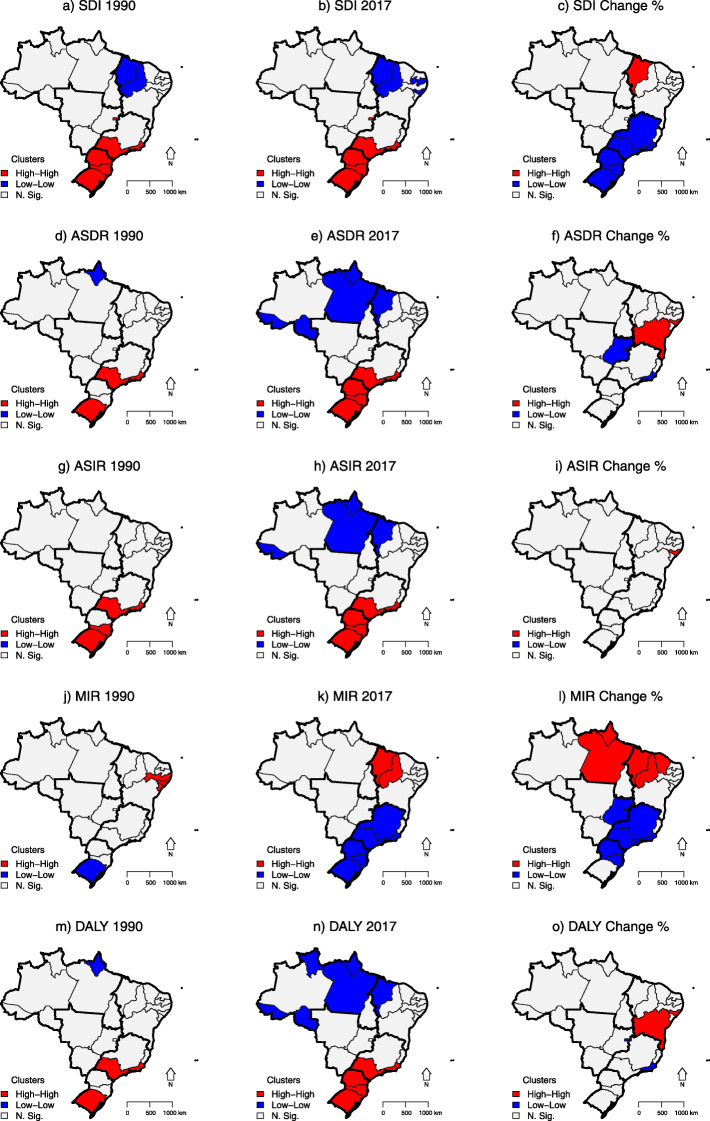
Lisa cluster maps of socio-demographic index (SDI), age-standardized female breast cancer death (ASDR), incidence (ASIR) and disability-adjusted life years (DALYs) rates and female breast cancer mortality-to-incidence (MIR) ratio, and percentual change. Brazil, 1990, 2017

Clusters of states with low SDI values were identified in the Northeast region and clusters of states with high SDI values were found in the Southeast and South regions. The clusters for mortality, incidence, and DALYs rates showed similar patterns, with low values in the North and Northeast regions and high values in the South and Southeast regions. On the other hand, when considering the percentage change of all these indicators, clusters of high values were observed in the North and Northeast regions, and of low values in the South and Southeast regions. However, MIR had different behavior, with clusters of low values in the South and Southeast regions, and high values in the North and Northeast regions, where there were also the lowest reductions in this indicator between 1990 and 2017 years, highlighting regional differences (Fig. 6).

In all the panel models, the Pesaran test was significant, indicating cross-sectional dependence and pointing the spatial models for adjusting the spatial correlation. The Hausman test was significant for models with ASDR, ASIR and DALYs as outcome variables, guiding the choice of fixed effect spatial panel models. As the model for MIR had non-significant Hausman test, the most appropriate choice was the spatial random effect model. The SDI had a positive association with incidence rate (coef = 30.38, *p* < 0.001; Table 5) and a negative association with MIR (coef = −0.212, *p* < 0.001; Table 5), showing no association with mortality rate.

**Table 5:** Panel models results for the association between socio-demographic index (SDI) and age-standardized female breast cancer death (ASDR), incidence (ASIR) and disability-adjusted life years (DALYs) rates and female breast cancer mortality-to-incidence (MIR) ratio in Brazilian states, 1990‑2017

Panel models	Coef. SDI	Std. error	*p* value
ASDR^a^			
Pooled model	20.390	1.194	< 0.001
Fixed effect model	−0.102	0.032	0.866
Random effect model	0.166	0.610	0.786
Spatial fixed effect lag model	0.079	0.560	0.888
Spatial random effect lag model	0.226	0.568	0.690
ASIR^b^			
Pooled model	86.500	2.685	< 0.001
Fixed effect model	55.815	1.363	< 0.001
Random effect model	56.147	1.365	< 0.001
Spatial fixed effect lag model	30.382	2.423	< 0.001
Spatial random effect lag model	30.707	1.221	< 0.001
DALY^c^			
Pooled model	520.538	33.847	< 0.001
Fixed effect model	26.597	17.385	0.127
Random effect model	32.640	17.498	0.063
Spatial fixed effect lag model	21.910	16.328	0.180
Spatial random effect lag model	25.628	16.496	0.120
MIR^d^			
Pooled model	−0.539	0.012	< 0.001
Fixed effect model	−0.834	0.009	< 0.001
Random effect model	−0.814	0.010	< 0.001
Spatial fixed effect lag model	−0.207	0.018	< 0.001
Spatial random effect lag model	−0.212	0.006	< 0.001

^a^Panel models for ASDR. Hausman test: chisq = 8.23; *p* = 0.004. Pesaran test: *z* = 15.23; *p* < 0.001. Selected model: spatial fixed effect lag model

^b^Panel models for ASIR. Hausman test: chisq = 13.99; *p* < 0.001. Pesaran test: *z* = 21.74; *p* < 0.001. Selected model: spatial fixed effect lag model

^c^Panel models for DALYs. Hausman test: chisq = 43.63; *p* < 0.0021. Pesaran test: *z* = 53.74; *p* < 0.001. Selected model: spatial fixed effect lag model

^d^Panel models for MIR. Hausman test: chisq = 2.04; *p* = 0.153. Pesaran test: *z* = 5.07; *p* < 0.001. Selected model: spatial random effect lag model

## Discussion

In Brazil, breast cancer mortality rate had a modest decrease (−4.45%) between 1990 and 2017 years, while the incidence rate has increased substantially (+39.99%). Furthermore, the MIR for breast cancer declined in the country between 1990 and 2017 years (−31.75%). The MIR, a proxy for survival rat e[5, 22], provides an alternative means to assess the burden of a disease. It is useful to identify inequities in cancer outcomes, enabling evaluation of cancer control programs, including cancer screening and treatment [29]. The SDI showed an increase in all Brazilian states in the same period, notably in less developed regions, showing a positive association with incidence rate and a negative association with MIR. Such findings reinforce the influence of socio-demographic improvement on the increase of breast cancer incidence. It also suggests an improvement in breast cancer survival in the period, which may be related to a broader access to diagnostic methods and recommended treatment. It is noteworthy, however, that the regions with the worst socioeconomic conditions showed a lower reduction of MIR, which is an indirect estimation of survival [5, 22]. The long-term survival of women with breast cancer has been increasing in Brazil, although it still shows lower rates than in higher-income countries. This increase in survival has been attributed to improvements in breast cancer treatment, and also to the increase in mammographic screening [30]. However, a study that analyzed data from hospital-based cancer registries in Brazil did not show a reduction in the late stage of breast cancer, even with the implementation of the screening national program in 2004 [31].

Brazilian studies of the trends in breast cancer mortality in the last decades show stability [8, 9] or even increase [10–14] in deaths due to breast cancer in Brazil. The variation in the findings is likely a function of the type of records included and the years considered. However, most studies used data from the Ministry of Health Information Systems (DATASUS) with the most recent follow-up, at most, until the year 2014. Even studies that found a trend of increased mortality are in accordance with the present findings with regard to the regional disparity of breast cancer mortality in Brazil. A study carried out with the mortality information system data from 1980 to 2009 found stability of breast cancer mortality in Brazil since 1994, with a tendency to increase in the North, Northeast, and Central West regions, stability in the South region and reduction in the Southeast region. The risk of death from breast cancer was at least twice as high in states such as São Paulo, Rio de Janeiro, Rio Grande do Sul, and the Federal District, which have higher Human Development Index (HDI), in comparison to the States of Alagoas, Maranhão, and Piauí [9]. In the present study, when considering the Brazilian regions and states, there was an increase in breast cancer mortality in the seven states, the less developed regions, while the greatest reductions were observed in the more developed regions. These findings were also observed in other Brazilian studies that worked on a subnational scale [8–13, 16, 32].

The increase in the breast cancer incidence in all Brazilian states between 1990 and 2017 years, with an even greater increase in the low-income states, point to the effect of the demographic and epidemiological change in Central and South America, with consequent change in disease determinants as reproductive and hormonal factors, and lifestyle factors, such as overweight and obesity, physical inactivity and alcohol consumption, and detection and mammographic screening [6, 13], mainly in less developed regions.

A recent study found that, between 1990 and 2015 years, mortality from breast cancer attributable to physical inactivity increased in Brazil (+0.77%; 95% U.I.: 0.27; 1.47) and decreased around the world (−2.84%; 95% U.I.: −4.35; −0.10) [7].

Although the DALYs rates for breast cancer remained stable in Brazil between 1990 and 2017 years, which may be related to the improvement in treatment and even in early detection, considering that there was an increase in breast cancer incidence in Brazil in the same period, subnational data showed an increase in DALYs rates mainly in states with lower SDI, especially those in the Northeast region. Although government strategies seem to be effective against the burden of breast cancer in Brazil [13], the rising burden of this disease in Brazilian regions with the lowest SDI (North and Northeast) leads increasingly to marked regional disparities. To meet this challenge, each country must identify its specific needs and priorities [18].

The SDI increased between 1990 and 2017 years both for Brazil as a whole and for all Brazilian states, which suggests a general improvement in the level of development in the country, which affected all regions. As SDI increases, cancers associated with lifestyle factors of more developed regions, such as breast cancer, are becoming more common [19]. On the other hand, the MIR declined between 1990 and 2017 years both for Brazil in general and for all states, which may be related to the increase in breast cancer incidence faster than mortality [5], or may also point to an implementation of an effective cancer control programs, including cancer screening [29].

An important limitation of the present study is the use of estimates of the indicators at national and mainly subnational scale, which can be influenced by variability of data sources, depending on the level of regional development, with higher quality in the more developed regions. However, the GBD 2017 study makes substantial efforts to enhance the comparability of results by applying corrections for under-registration and garbage code redistribution algorithms [19].

In addition, the indicators estimated in this study, based on data from the GBD 2017 study, were very close to those estimated in other studies that used data from the national information systems, which reinforces the validity of our results.

## Conclusion

This study found that there are inequalities in breast cancer outcomes among Brazilian states and regions. These findings may help to guide public policy priorities in the country, as well as enable an evaluation of the breast cancer control program.

The study included a follow-up of the burden of breast cancer over an extended period of time (from 1990 to 2017), which allowed a more valid prediction about breast cancer trends in Brazil.

Periodic evaluations using consistent indicators can help evaluate the success of breast cancer prevention, diagnosis, and treatment in Brazil.

## Supplementary information

**Additional file 1:** Figure S1. Brazilian Regions and States.

**Additional file 2:** Figure S2. Temporal distribution of age-standardized female breast cancer death (ASDR) and incidence (ASIR) rates in Brazil and Brazilian States, 1990-2017.

**Additional file 3:** Figure S3. Temporal distribution of socio-demographic index (SDI) and female breast cancer mortality-to-incidence (MIR) ratio in Brazil and Brazilian States, 1990-2017.

**Additional file 4:** Figure S4. Temporal distribution of female breast cancer disability-adjusted life years (DALYs) rate, 1990-2017.

## Data Availability

The datasets supporting the conclusions of this article are publicly available online in the official website of the Institute of Health Metrics and Evaluation: http://ghdx.healthdata.org/gbd-results-tool.

## Abbreviations

ASDR: Age-standardized breast cancer death
ASIR: Age-standardized breast cancer incidence
DALYs: Disability-adjusted life years
DATASUS: Brazilian Ministry of Health Information Systems
IBGE: Brazilian Institute of Geography and Statistics
IHME: Institute for Health Metrics and Evaluation
GBD: Global burden of disease
HDI: Human Development Index
MIR: Mortality-to-incidence ratio
SDI: Socio-demographic index
UI: Uncertainty interval
