# Supplementary figures and images for: Inequalities in the burden of female breast cancer in Brazil, 1990–2017
Source: Popul Health Metr. 2020 Sep 30;18(Suppl 1):8. doi: 10.1186/s12963-020-00212-5 (PMC7525962; doi:10.1186/s12963-020-00212-5)

# Brazilian Regions and States

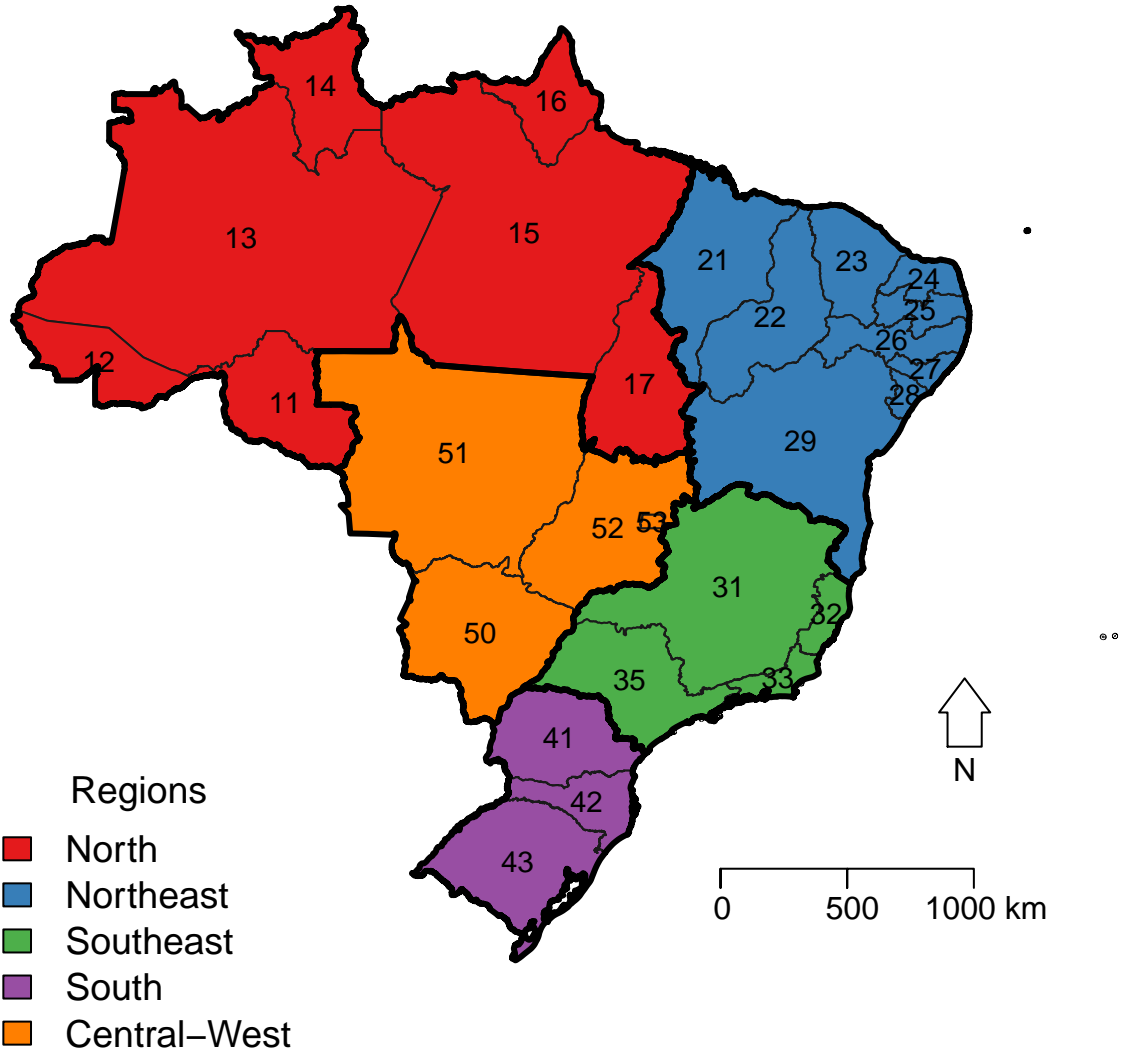

Numbers on the map are the IBGE codes for States

Supplement: Supplementary file 1 — Additional file 1: Figure S1. Brazilian Regions and States. [file 12963_2020_212_MOESM1_ESM.pdf]

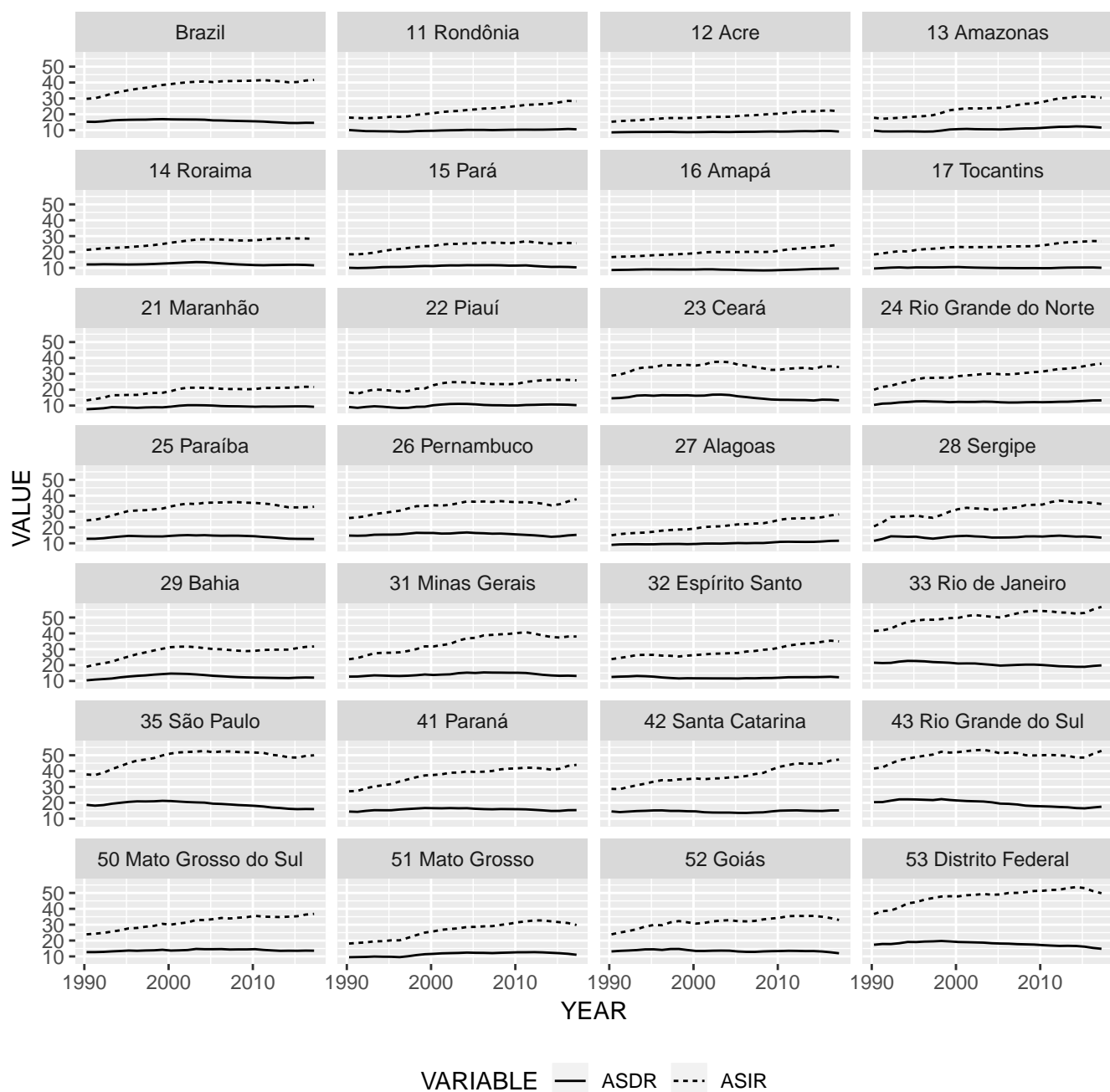

Supplement: Supplementary file 2 — Additional file 2: Figure S2. Temporal distribution of age-standardized female breast cancer death (ASDR) and incidence (ASIR) rates in Brazil and Brazilian States, 1990-2017. [file 12963_2020_212_MOESM2_ESM.pdf]

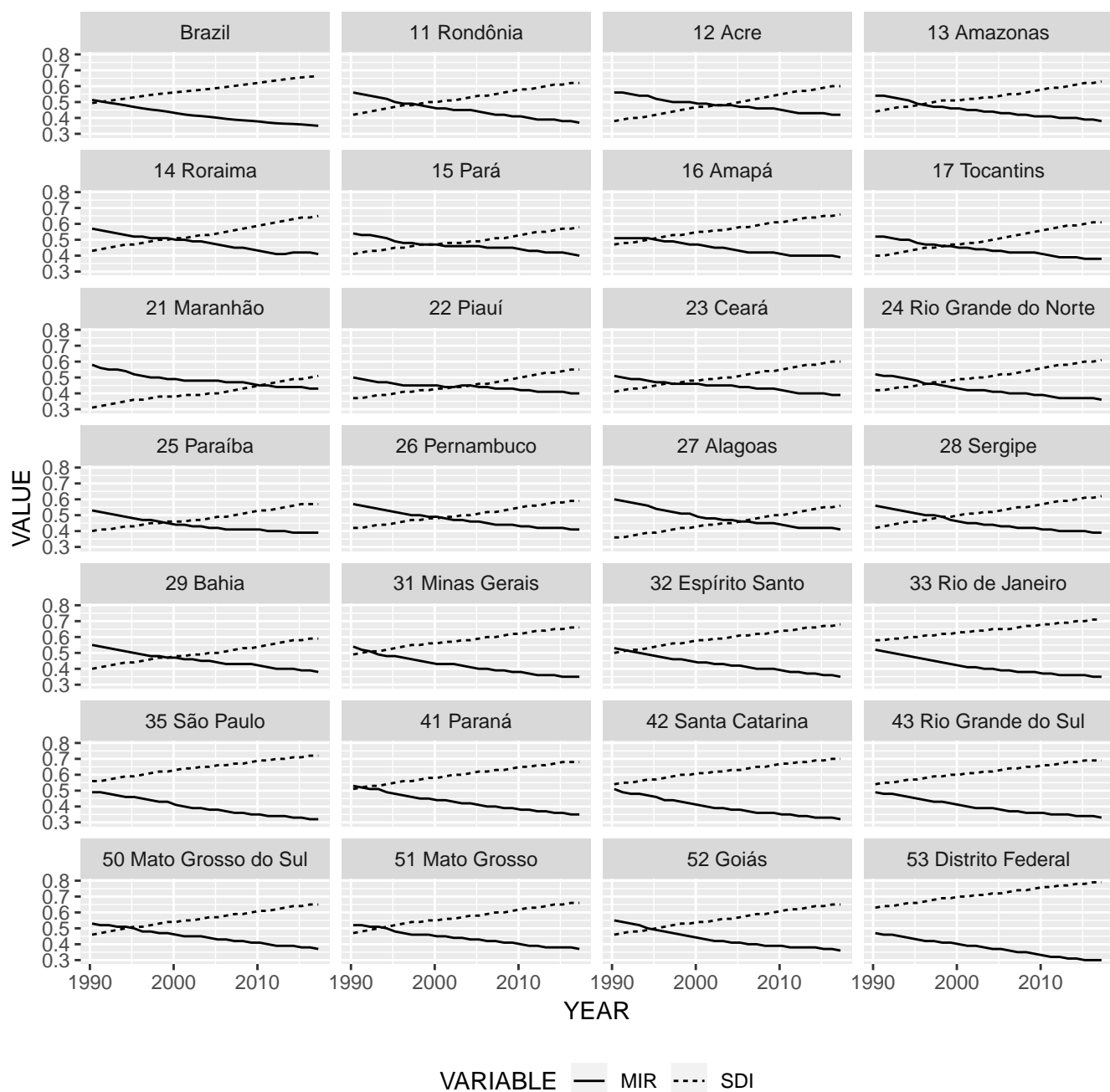

Supplement: Supplementary file 3 — Additional file 3: Figure S3. Temporal distribution of socio-demographic index (SDI) and female breast cancer mortality-to-incidence (MIR) ratio in Brazil and Brazilian States, 1990-2017. [file 12963_2020_212_MOESM3_ESM.pdf]

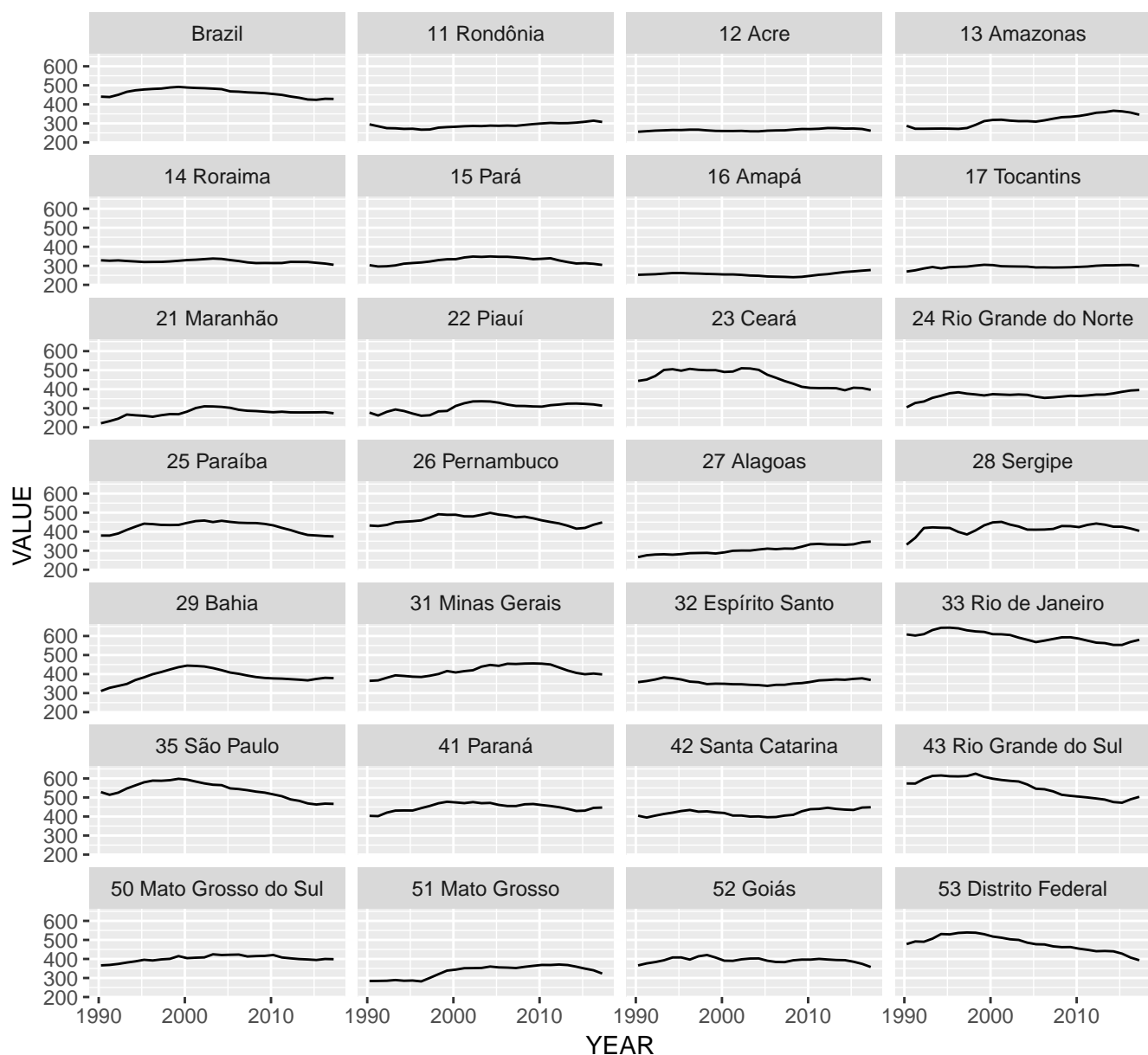

Supplement: Supplementary file 4 — Additional file 4: Figure S4. Temporal distribution of female breast cancer disability-adjusted life years (DALYs) rate, 1990-2017. [file 12963_2020_212_MOESM4_ESM.pdf]
